# Dilated Virchow Robin spaces in multiple sclerosis – a generalised marker of disease?

**DOI:** 10.1016/j.ebiom.2023.104708

**Published:** 2023-07-07

**Authors:** Lucy Vivash

**Affiliations:** a Department of Neuroscience, Central Clinical School, Monash University, Melbourne 3004, Australia; b Department of Neurology, Alfred Hospital, 55 Commercial Road, Melbourne 3004, Australia

Virchow Robin Spaces (VRS), also known as perivascular spaces surround cerebral blood vessels and play an important role in fluid exchange between the blood, cerebrospinal fluid and interstitium. Enlarged VRS are commonly observed in the brain in both health and disease. Whilst there is a body of evidence to suggest greater VRS burden is associated with cardiovascular risk factors (hypertension, higher BMI^1^), it has yet to be established how (and if) they contribute to disease pathogenesis. Moreover, there is very little evidence to associate enlarged VRS with clinical measures of disease severity, either cross-sectionally or longitudinally across the majority of neurological and neurodegenerative diseases where VRS have been observed.^2^^,^^3^

In multiple sclerosis (MS) many studies of enlarged VRS have found associations with lesion volume and disease severity, however a meta-analysis of 9 studies (>450 patients) failed to replicate these associations, demonstrating increased VRS count and volume in patient with MS compared to controls but no associations with disease severity or progression, supporting the use of VRS as a diagnostic or prognostic tool in MS.^4^ The strength of these findings remain under consideration due to limitations in the methodology of identifying and quantifying VRS number and volume, with the majority of these previous studies utilising gross scales of VRS burden rather than quantification of number or volume. More recent studies in substantial patient populations using higher resolution images have quantified VRS demonstrating associations with differing combinations of disease severity and duration, lesion volume, cortical atrophy and diffusion metrics.^5^, ^6^, ^7^ Improvements in MRI resolution and methods for detecting VRS are increasing the detection of VRS on clinical scans, the clinical significance of which has yet to be determined. It is also postulated that it is the not the presence of VRS, but dilated VRS, that are indicative of disease, however evidence for this is limited.

The study by Ineichen and colleagues reported an extensive study of VRS in patients with MS.^8^ Building on previous studies, they specifically differentiated dilated VRS (>2 mm) from undilated VRS, assessing the spatial distribution in relation to MS lesions, brain atrophy and clinical severity. Whilst increased numbers of dilated and undilated VRS were observed, they did not correlate with disease staging or cognitive function. In a small subset with repeated MRIs changes in clinical stage or lesion load did not associate with changes in the number of dilated VRS. Post-mortem histology demonstrated dilated VRS did not colocalise with demyelination, axonal damage or microglial/macrophage activation but with arteries and evidence of arterial disease. Together these results suggest dilated VRS are associated with cardiovascular disease rather than MS pathology.

Overall the study demonstrates that the number of dilated VRS are highly variable in both patients and healthy controls, even when controlling for cardiovascular risk factors. Despite a generous definition, the number of dilated VRS detected across all participants studied was low. Time of day and sleep are other variables not controlled for in the current study which are thought to be associated with the presence of dilated VRS. Diurnal variation of VRS is well established, with dilation of VRS thought to occur during sleep.^9^ The timing of the MRI relative to wake and sleep deprivation both contribute to the size and visibility of VRS.^1^

Another major limitation of the current study is the availability of data on disease-modifying treatments (DMTs) used by the patients and the time course of DMT intervention relative to the timing of MRIs. Given the high rate of DMT use in both MS cohorts the lack of association with lesion load or other markers of active neuroinflammation is not surprising.

The study demonstrates elevated dilated VRS in patients with MS. However, the limited correlation with other measures of disease severity opens the possibility that dilated VRS may be a non-specific marker of disease, be it neurological, cardiovascular or a combination of the two. There are many contributing factors to the presence of dilated VRS, more comprehensive studies controlling for these many factors are needed not only in MS, but across neurological and neurodegenerative diseases to better understand the role of VRS in disease pathogenesis and presentation.

## Contributors

LV was the only contributor to this commentary.

## Declaration of interests

The author does not have any conflict of interest to disclose.

## References

[1] Barisano G., Sheikh-Bahaei N., Law M., Toga A.W., Sepehrband F. (2021). Body mass index, time of day and genetics affect perivascular spaces in the white matter. J Cereb Blood Flow Metab.

[2] Moses J., Sinclair B., Schwartz D.L. (2022). Perivascular spaces as a marker of disease severity and neurodegeneration in patients with behavioral variant frontotemporal dementia. Front Neurosci.

[3] Zhang C., Xu K., Zhang H. (2023). Recovery of glymphatic system function in patients with temporal lobe epilepsy after surgery. Eur Radiol.

[4] Granberg T., Moridi T., Brand J.S. (2020). Enlarged perivascular spaces in multiple sclerosis on magnetic resonance imaging: a systematic review and meta-analysis. J Neurol.

[5] Kolbe S.C., Garcia L.M., Yu N. (2022). Lesion volume in relapsing multiple sclerosis is associated with perivascular space enlargement at the level of the basal ganglia. AJNR Am J Neuroradiol.

[6] Carotenuto A., Cacciaguerra L., Pagani E., Preziosa P., Filippi M., Rocca M.A. (2022). Glymphatic system impairment in multiple sclerosis: relation with brain damage and disability. Brain.

[7] Liu X.Y., Ma G.Y., Wang S. (2022). Perivascular space is associated with brain atrophy in patients with multiple sclerosis. Quant Imaging Med Surg.

[8] Ineichen B.V., Cananau C., Platten M. (2023). Dilated Virchow-Robin spaces are a marker for arterial disease in multiple sclerosis. EBioMedicine.

[9] Aribisala B.S., Valdes Hernandez M.D.C., Okely J.A. (2023). Sleep quality, perivascular spaces and brain health markers in ageing - a longitudinal study in the Lothian birth cohort 1936. Sleep Med.

